# Bioactive Peptides From the *Cucurbitaceae* Family: From Identification and Health‐Promoting Functions to Industrial Utilization

**DOI:** 10.1002/fsn3.72338

**Published:** 2026-09-16

**Authors:** Himani R. Kekiri Obada Gamage, Mahya Bahmani, Utpal Bose, Mekhala Vithana, Michelle L. Colgrave, Angéla Juhász

**Affiliations:** ^1^ School of Science Edith Cowan University Joondalup Western Australia Australia; ^2^ Australian Research Council Centre of Excellence for Innovations in Peptide and Protein Science Edith Cowan University Joondalup Western Australia Australia; ^3^ CSIRO Agriculture and Food Floreat Western Australia Australia; ^4^ CSIRO Agriculture and Food St. Lucia Queensland Australia

**Keywords:** bioactive, *Cucurbitaceae*, industrial utilization, plant peptides, proteomics

## Abstract

*Cucurbitaceae* is a major plant family within the kingdom *Plantae*, encompassing popular food crops such as zucchini, squash, pumpkins, and melons. With the growing interest in discovering novel bioactive peptides (BPs) from various plant sources, *Cucurbitaceae* crops have emerged as a popular candidate for isolating nutrient‐enriched peptides due to their diverse applications in the nutritional and pharmaceutical sectors, with experimentally evidenced antihypertensive, antidiabetic, antimicrobial, and anticancer properties. However, current investigations are limited to a few economically important species and are seed‐centric within this family, with limited in vivo and clinical validations for their in vitro functional properties. In this review, we have summarized the peer‐reviewed original proteomic research and reviews, written in English, reported to date on peptide production from protein extraction to hydrolysis, identification, and functional characterization, as well as their industrial translation, excluding those without a standard functional validation. This work discusses and evaluates the applied protocols, limitations, and challenges in BP discovery and industrial utilization, highlighting understudied cucurbits as a potential source of novel BPs and insights into broadening future research scopes and providing opportunities aligned with plant‐derived peptide discovery.

## Graphical abstract

From fruit to function: Cucurbitaceae‐derived peptide production, bioactive potential screening and industrial applications.

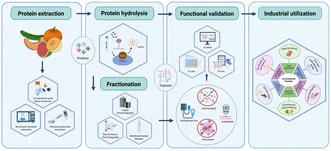

**Abbreviations**
AA: amino acid
ABTS: 2,2′‐azino‐bis (3‐ethylbenzothiazoline‐6‐sulfonic acid)
ACE: angiotensin‐converting enzyme
AMPs: antimicrobial peptides
BPs: bioactive peptides
ddH_2_O: double‐distilled H_2_O
DH: degree of hydrolysis
DPPH: 2,2‐diphenyl‐1‐picrylhydrazyl
E/S: enzyme–substrate ratio
GI: gastrointestinal
MW: molecular weight
ROS: reactive oxygen species
RP‐HPLC: reverse‐phase high‐performance liquid chromatography
SEC: size‐exclusion chromatography

## 1 Introduction

Bioactive peptides (BPs) are short amino acid chains, typically comprising 2–20 residues, that exert measurable physiological or biological effects in living cells (Akbarian et al. 2022). These peptides can be divided into two major groups: endogenous and exogenous BPs. The endogenous BPs are produced internally as cryptides or native peptides in different tissues of the organism and play essential physiological functions as signaling molecules for cellular activities (Tavormina et al. 2015). Exogenous BPs are generated externally through dietary sources or supplements. Plant sources like fruits and vegetables, animal sources like meat or milk, fungi and fermented products are potential sources of BPs with health benefits, extensively studied elsewhere (Aguilar‐Toala et al. 2017; Ashaolu and Yupanqui 2018; Bütikofer et al. 2007; Cicek 2022; Ozuna and Leon‐Galvan 2017; Sachin et al. 2021; Silva et al. 2017; Wang and Ng 2003; Ahmed et al. 2024). Although BPs from plants and animals have numerous potential applications in human health, the complete spectrum of their diversity and functions remains largely unexplored.

Plants play an important role in the human diet as a source of nutrition and as natural repositories of diverse BPs. They are also rich in small‐molecule bioactive compounds, including carotenoids, phenolic compounds, phytosterols, and biogenic amines (Samtiya et al. 2021; Šaponjac et al. 2016). Plant‐based BPs are generally more accessible, abundant, and culturally acceptable, and they present lower toxicity and production costs than peptides of animal origin (Fan et al. 2022). Plant‐derived BPs show various biological and physiological functions based on their AA composition and size, including antimicrobial, antidiabetic, and antihypertensive activity via angiotensin‐converting enzyme (ACE) inhibitory function, cytomodulatory function, and immunomodulatory function (Korhonen and Pihlanto 2006).

Plant BPs originate from either precursor‐derived or non‐precursor‐derived biosynthetic routes. In the precursor‐derived pathway, bioactive peptides are cleaved from larger proteins, and those may be functional or non‐functional (Tavormina et al. 2015). Most plant‐derived BPs are synthesized via this precursor‐derived ribosomal route, producing either unmodified or post‐translationally modified ribosomally synthesized peptides (RiPPs) (Tavormina et al. 2015). Conversely, non‐precursor‐derived peptides can arise from small open reading frames (smORFs) within non‐coding RNAs, such as long non‐coding RNAs (lncRNAs) and primary miRNAs (pri‐miRNAs) (Sruthi et al. 2022; Pan et al. 2018). In addition, peptides classified as non‐ribosomal peptides (NRPs) are synthesized by non‐ribosomal peptide synthetases without the involvement of ribosomes or RNAs (Miller and Gulick 2016). These NRPs show potent antimicrobial properties and are produced mainly by fungi and bacteria, including the symbionts of higher eukaryotes; however, they are relatively less characteristic in plants and animals (Soltani 2016).

*Cucurbitaceae* is among the most diverse plant families within the *Plantae* kingdom, comprising around 1000 species. These crops were domesticated in pre‐Columbian times, resulting in larger fruit size, reduced bitterness, increased sugar and carotenoid content, and fewer physical defenses than their wild ancestors (Chomicki et al. 2020). Since their domestication, many cucurbits have been utilized as popular food sources and recognized for their proven medicinal values, including antidiabetic, antioxidant, and purgative properties derived from high levels of carotenoids, terpenoids, and other polyphenolic compounds (Rolnik and Olas 2020). As reported by Enujiugha et al. (2019), the pulps and seeds of *Cucurbita maxima* and *Cucurbita mixta* fulfill more than 20% of the recommended dietary allowances (RDA) of several essential amino acids, including leucine, isoleucine, methionine, lysine, phenylalanine, and valine, for adults. Moreover, according to WHO recommendations (World Health Organization 2023), consuming more than 400 g of fruits and vegetables per day is beneficial against certain types of cancers and cardiovascular diseases, where *Cucurbitaceae* fruits can be a potential option. Cucurbit genera such as *Cucurbita*, *Cucumis*, and *Citrullus* are considered the most utilized in the *Cucurbitaceae* family with worldwide economic importance. In 2023, global pumpkin, squash, and gourd production exceeded 23 million tons, whereas cucumber and gherkin production was approximately 98 million tons, according to FAO (2025). In addition, many other members in the genus *Momordica*, *Lagenaria*, *Luffa*, *Benincasa*, and *Trichosanthes* have been identified as minor crops with local economic importance in African, Asian, and South American regions (Chomicki et al. 2020).

With recent advances in BP discovery, *Cucurbitaceae* crops have been identified as a valuable source of plant‐derived BPs in many reported studies (Li et al. 2024; Lin et al. 2024; Shahin‐Kaleybar et al. 2020; Yuan et al. 2008). However, existing knowledge is limited to a few commonly utilized *Cucurbitaceae* crops with limited or no in vivo and clinical validations, and studies are primarily focused on seed proteins, overlooking other tissues, such as leaves, roots, and fruit peels, which could also be potential sources of novel BPs. Furthermore, the influence of post‐harvest factors, including temperature, relative humidity, air circulation, and ethylene exposure, on the qualitative and quantitative variation of BP in these crops remains to be explored. Key knowledge gaps persist in terms of the stability, bioavailability, and safety of cucurbit peptides, and only a few studies have considered industrial scale‐up, regulatory approval, or consumer acceptance, compared with peptides derived from cereals and legumes.

This review, therefore, aims to consolidate current knowledge on *Cucurbitaceae*‐derived bioactive peptides, highlight their nutritional and therapeutic potential, summarize state‐of‐the‐art approaches for peptide discovery and characterization, and identify major research and application gaps to guide future study and industrial translation. For this purpose, we have summarized the peer‐reviewed original research and comprehensive/critical reviews, written in English, reporting on *Cucurbitaceae* peptides from 1981 to 2025. The literature search was conducted using PubMed, Scopus, and Google Scholar databases, with search terms including combinations of phrases such as “*Cucurbitaceae* peptides,” “Bioactive properties of cucurbits,” “Bioactive peptides from *Cucurbitaceae* seed proteins,” “Industrial utilization of cucurbit peptides” among other related terms specific to the family *Cucurbitaceae*. Studies were included if they reported standard functional validations of the identified peptides; studies without such validations were excluded.

## 2 Production and Identification of Bioactive Peptides

**FIGURE 1:**
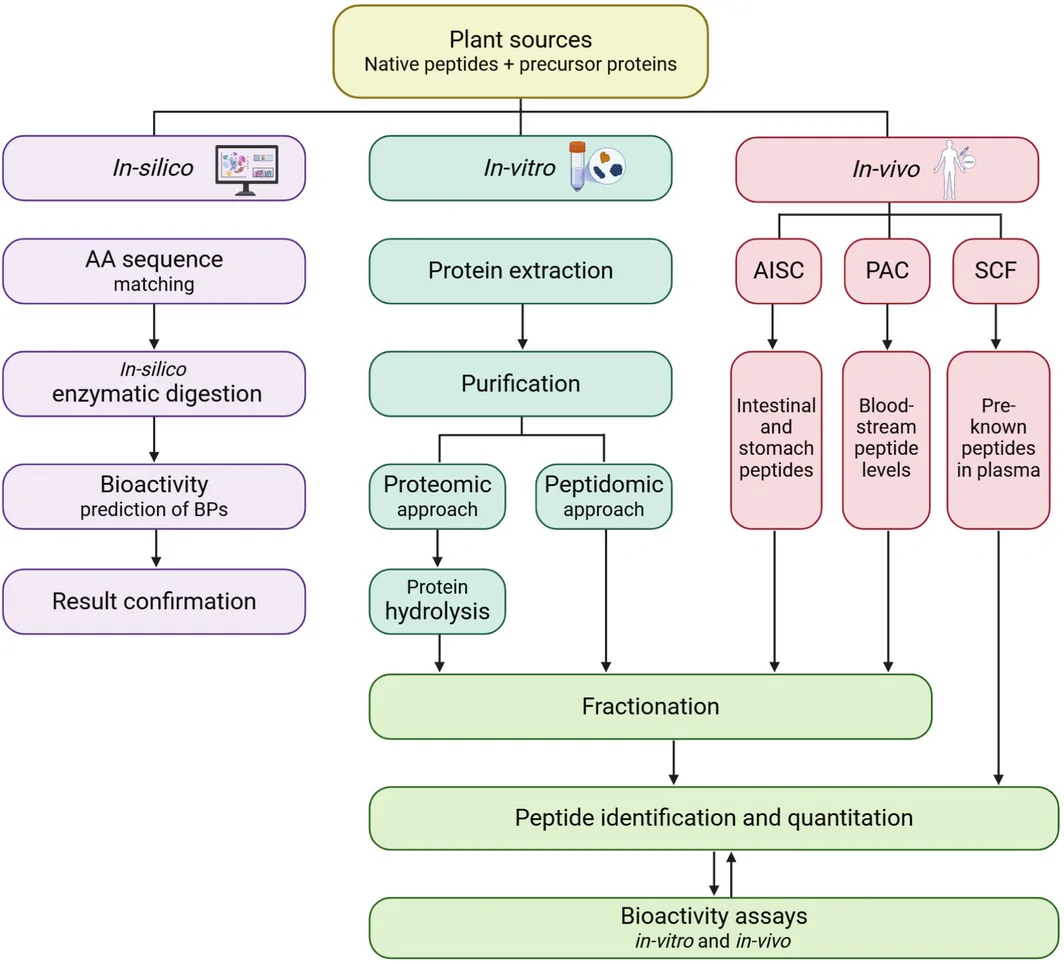
Key proteomic workflows for the plant‐derived BP discovery and production. AISC, aspiration of intestinal and stomach content method; PAC, post‐absorption characterization method; SCF, standard curve‐fitting method. The image was created using BioRender.

Bioactive peptide discovery traditionally relies on analytical, bioinformatics, or integrated approaches combining both methods. The analytical approach involves assessing the bioactivity of isolated protein hydrolysates through in vitro, ex vivo, and/or in vivo functional evaluations complemented with proteomic and peptidomic approaches. The in silico bioinformatics workflow identifies BPs based on the presence of a sequence embedded within a parent protein or the genomic sequences, as the plant proteomes and genomes are better studied than plant peptidomes (Daliri et al. 2017; Dos Santos‐Silva et al. 2020). Figure 1 summarizes the generally applied in silico, in vitro, and in vivo workflows for BP production.

There are two fundamental BP discovery approaches: native peptidomics and bottom‐up proteomics. The native peptidomics approach targets endogenous peptides occurring naturally in their intact forms. Conversely, bottom‐up proteomics focuses on extracting proteins from biological matrices, followed by enzymatic digestion to produce peptides, as protein fragments prior to MS analysis (Di Meo et al. 2016). These approaches have gained traction over the last decade for their capacity to identify BPs for industries like pharmaceuticals, food, and agriculture.

Following the discovery of BPs from plant sources, their functional properties are often validated through in vitro and in vivo experiments. The production of BPs can also be achieved using in vitro methods, for instance via enzymatic methods that use commercially available proteolytic enzymes or as a result of microbial fermentation (Ozuna and Leon‐Galvan 2017; Bayat et al. 2014; Dia and Krishnan 2016). Most of the existing cucurbit peptide studies have used this approach to identify and characterize BPs. Ex vivo‐based approaches are also common in peptide discovery, where biologically relevant organs/tissues are used for the identification of peptides and often perform without in vitro or after in vitro production and characterization. However, there are no reported ex vivo‐based *Cucurbitaceae* peptide discovery studies.

According to Barati et al. (2020), BP production using the in vivo method is significantly more complex than in vitro and ex vivo, as it requires the separate identification of exogenous peptides from dietary or environmental sources and of endogenous peptides within the host organism. The aspiration of intestinal and stomach content (AISC) method is a key in vivo approach and provides more realistic information regarding peptide production and characterization compared to in vitro methods; however, it is considered invasive. Similarly, as in in vitro and ex vivo, in AISC, the peptide absorptivity aspect is not covered. Conversely, post‐absorption characterization (PAC), which is another in vivo approach, can be used as a BP production and characterization technique, which involves differential identification of endogenous and exogenous peptides in the bloodstream, covering the BP absorptivity aspect as well (Barati et al. 2020). The standard curve‐fitting (SCF) method is also an in vivo technique used in BP identification and in measuring predetermined peptide levels in plasma, but not for BP production or characterization. Though these in vivo methods have the potential to produce BPs, the processes are often invasive and complex, making them more characterization approaches rather than BP production. There are only a few reported studies based on in vivo cucurbit peptide functionality characterization reported to date (Priyanto et al. 2015; Nkosi et al. 2006a), emphasizing the need for more in vivo and clinical evidence‐based studies.

Recent advances in computational biology have accelerated BP discovery through in silico methods. These approaches predict biologically active peptide sequences by mining databases and protein datasets to identify segments with known or putative functionality (Khaket et al. 2014; Rani et al. 2018; Senadheera et al. 2022). Databases such as BIOPEP‐UWM, PepBank, and BioPD contain extensive repositories of validated peptide sequences and biological properties, serving as reference datasets for predictive analyses (Minkiewicz et al. 2019; Shi et al. 2004; Shtatland et al. 2007). Additionally, in silico proteolytic enzyme prediction tools, like Peptide Cutter, BIOPEP, and Enzyme Predictor, can be used to identify and select candidate sequences for subsequent chemical synthesis, which can then be tested via in vitro and in vivo studies (Senadheera et al. 2022; Tu et al. 2018).

Despite the efficiency of in silico approaches, discrepancies may occur between predicted and experimentally identified peptides due to sequence variations and/or post‐translational modifications (PTMs) of otherwise evolutionarily conserved proteins across different species. These modifications, including proteolytic cleavage, disulfide bond formation, glycosylation, amidation, and phosphorylation, are often overlooked in computational models and can significantly alter the biochemical properties and biological activity of peptides (Barati et al. 2020).

Liang et al. (2021) used an in silico pathway to identify a novel tripeptide (IAF) from *Cucurbita moschata* (pumpkin) seeds that possessed ACE‐inhibitory activity. According to molecular docking studies and molecular dynamics simulations, the peptide binds to key residues in the active site of the ACE enzyme, forming a stable enzyme‐peptide complex (IAF‐ACE) through hydrogen bond interactions and chelation with Zn^2+^, thereby inhibiting its enzyme activity. Similarly, Wong et al. (2021) revealed the use of an in silico BP discovery approach, combined with molecular docking to identify anti‐COVID‐19 peptides from *C. maxima* seeds. They subjected pumpkin seed storage proteins to in silico gastrointestinal (GI) digestion, obtaining 197 peptide fragments and screened for the peptides with the highest GI absorption using molecular docking simulations. The detected seed protein hydrolysates were found to contain a pumpkin‐derived dipeptide, PW (Pro‐Trp), that targets the SARS‐CoV‐2 spike protein, main protease, and papain‐like protease. However, the findings have not been experimentally validated in vivo or in vitro to date, as is necessary to confirm their bioactivity and physiological relevance.

### 2.1 Proteins and Peptides From *Cucurbitaceae* Tissues

The protein content composition of *Cucurbitaceae* species displays substantial qualitative and quantitative diversity across genera, species, and even plant tissues. Protein content varies widely between seeds and fruits, and other organs, due to differing physiological roles and storage functions (Ruwanthika et al. 2023). For instance, Nguyen et al. (2019) reported that dried watermelon rind contains 19.1% crude protein. However, as reported by Raihana et al. (2015), on a dry‐weight basis, watermelon seeds contained crude protein ranging from 25.2% to 37%. These differences reflect the abundance of seed‐specific storage proteins, such as albumins, prolamins, and globulins, in seeds compared to other tissues, making them an attractive and widely studied substrate for BP isolation.

Protein and peptide extraction from plant tissues poses unique challenges due to the structural complexity of the rigid cell walls and cellular membranes (González‐García et al. 2014; Soares et al. 2015). Effective extraction protocols aim to maximize the extraction of proteins from experimental tissues for downstream analysis. Selecting an optimal protein extraction method depends on the total protein content of the source, presence of lipids and polysaccharides, physicochemical properties, cost, reproducibility, downstream applications, and final yield (Olivares‐Galvan et al. 2020). Different protein extraction methods and solvent compositions have been tested for BP discovery. Table 1 summarizes the different techniques used in protein extraction from *Cucurbitaceae* crops.

**TABLE 1:** Different protein extraction methods used in extracting proteins and peptides from cucurbitaceous crops.

*Cucurbitaceae* crop	Tissue	Extraction method	Extraction conditions	Yield%	References
*Benincasa hispida*	Seeds	Solid–liquid extraction	Extracted in acetone for 16 h at normal room temperature, then suspended in the extraction buffer of 20 mM potassium phosphate buffer, pH 6.5, 5 mM EDTA (ethylenediaminetetraacetic acid), and 1 mM DTT (dichloro‐diphenyl‐trichloroethane) for 3 h, and centrifuged at 10,000 rpm for 10 min at 4°C	Not reported	Sharma et al. (2014)
*Citrullus colocynthis*	Aerial parts	Solid–liquid extraction	Extracted into dichloromethane/methanol in a 1:1 (v/v) ratio by continuous agitation overnight at 25°C. Followed by liquid–liquid fractionation with ddH_2_O	10.75	Shahin‐Kaleybar et al. (2020)
*Citrullus lantanus*	Seeds	Solid–liquid extraction	NaOH at pH 12, 18°C, followed by protein precipitation using HCl at pH 4	Not reported	Arise et al. (2016)
*Citrullus lantanus*	Seeds	Solid–liquid extraction	NaOH at pH 10, 40°C, followed by the acidic precipitation of proteins by HCl at pH 4.5	35.15–38.27	Wani et al. (2011)
*Cucumis maderaspatensis*	Seeds	Solid–liquid extraction	20 mM phosphate buffer at pH 7.2 containing 50 mM sodium chloride (extraction buffer) and stirring for 6–8 h at 4°C. Followed by centrifuging at 10,000 rpm for 20 min	Not reported	Sachin et al. (2021)
*Cucurbita maxima*	Seeds	Solid–liquid extraction	NaOH at pH 11, at 50°C for 1.5 h. Then centrifuged at 9600 rpm for 30 min and followed by protein precipitation with 1 N HCl at pH 5.3	75.26–80.89	Lin et al. (2024)
*Cucurbita pepo*	Seeds	Solid–liquid extraction	Extracted with water (pH 10). Followed by protein precipitation at pH 5 and centrifugation	Not reported	Nkosi et al. (2006b)
*Momordica charantia*	Seeds	Solid–liquid extraction	Aqueous ethanol at 30°C for 15 min. Then centrifuged at 15,800 × *g* for 10 min. Followed by adding acetone to the supernatant and placing it at −80°C for 2 h. Centrifugation to recover the precipitated proteins	Not reported	Dia and Krishnan (2016)
*M. charantia*	Fruit pulp	Solid–liquid extraction	4°C with ice‐cold acid–ethanol (0.05 M H_2_SO_4_, 60% ethanol) and followed by pH adjusted to 3 using an ammonia solution. Then, acetone was used for protein precipitation	Not reported	Yibchok‐anun et al. (2006)
*Momoridca charantia*	Leaves	Vacuum‐assisted solid–liquid extraction	Cold 0.05 M sodium acetate buffer, pH 5.0 for 8 min. A vacuum was then applied (0.08 MPa) for 10 min. Then centrifugation at 6500 × *g* for 10 min at 4°C	Not reported	Zhang et al. (2015)
*Momordica cochinchinensis*	Seeds	Ultra‐sound‐assisted extraction (UAE)	Extracted into deionized water using 50 kHz ultrasounds at 40°C temperature and 250 W working power	Not reported	Le et al. (2018)

Solid–liquid phase extraction is the most conventional method for protein extraction from plant sources (Kanbargi et al. 2017; Meshginfar et al. 2018). This approach selects solvents based on the protein's isoelectric points, which are typically low for plant‐derived proteins (Olivares‐Galvan et al. 2020). For example, Wani et al. (2011) extracted seed proteins from *Citrullus lantanus* (watermelon) using 0.5 M NaCl and NaOH (pH 10, 40°C), followed by the acidic precipitation of proteins by HCl (pH 4.5), which resulted in protein yields ranging from 35.2% to 38.3%. Furthermore, Arise et al. (2016) reported watermelon seed protein extraction using NaOH (pH 12, 18°C), followed by protein precipitation using HCl (pH 4), and the protein yield of the isolate was 18.9%, suggesting lower alkalinity and high temperature conditions could be beneficial in enhancing the yield. Shahin‐Kaleybar et al. (2020) used dichloromethane and methanol (1:1, v/v) ratio to extract peptides from *Citrullus colocynthis* (colocynth) aerial parts, followed by liquid–liquid fractionation with double‐distilled H_2_O (ddH_2_O). According to the study, after the fractionation step, 430 mg of crude dried material was obtained from the peptide‐rich aqueous phase, derived from 400 g of colocynth tissues. Although this solid–liquid phase conventional protein/peptide extraction method is widely used and accepted at the industrial scale, it has some drawbacks, as it has high solvent consumption, is time‐consuming, has low selectivity, and results in lower yields, necessitating additional downstream purification steps (Soquetta et al. 2018).

Emerging techniques such as ultrasound‐assisted extraction (UAE) have gained attention for their higher efficiency and ability to recover bioactive proteins, peptides, and small molecules such as phenolics (Soquetta et al. 2018). UAE treatments (20 kHz–100 MHz) release intracellular components by accelerating heat and mass transfer within the system to disrupt the plant cell (Soquetta et al. 2018). Furthermore, Wen et al. (2019) found that slit divergent ultrasound (SDU) pretreatment at 20/28 kHz can increase the antioxidant potential and stability of watermelon seed protein hydrolysates compared to flat divergent ultrasound (FDS)‐treated and untreated fractions.

Microwave‐assisted extraction (MAE) is also a modern method used to extract plant proteins. This approach uses microwaves to accelerate energy transfer and reduce the thermal gradient to facilitate solvent penetration into the solid matrix and protein release (Navaf et al. 2023). According to Zaky et al. (2021), MAE is reproducible, cost‐effective, and can reach higher temperatures in a short time compared to other conventional heating methods. Liu et al. (2017) reported a combined method of ultrasound and microwave, with polyethylene glycol‐based deep eutectic solvent as the medium for extracting proteins from *C. moschata* (pumpkin) seeds. The protein yield reached 93.95% ± 0.23% under optimized conditions (43°C, 140 W, 28 g/mL solid‐to‐liquid ratio, 4 min). However, no study to date has directly compared this method's extraction efficiency against the conventional solid–liquid extraction using *C. moschata* seeds specifically. In the study by Lin et al. (2024) the protein yields of *C. maxima* (pumpkin) seeds were reported as 40.27% and 44.14% after 12 h of hot air drying and 54 h of freeze drying, followed by the conventional alkaline extraction and acidic precipitation. Although this suggests the synergistic extraction method with UAE and MAE could be fast and efficient compared to the conventional solid–liquid extraction, the two studies differ in experimental conditions, and further studies are required to confirm this comparison.

Pre‐extraction treatments such as drying, grinding, and defatting further enhance protein extraction efficiency. Grinding increases the solid particles' surface area, thereby enhancing contact with the extraction solvent (Naviglio et al. 2019). Defatting is a common practice when extracting proteins from seeds to minimize lipid interference during extraction. For instance, as reported by Siddeeg et al. (2015), n‐hexane at a ratio of 1:5 (w/v) has been used for semi‐defatting *Cucumis melo* (muskmelon) seed powder, followed by Soxhlet extraction to fully defat the powder before protein extraction. Moreover, Fan et al. (2014) reported defatting using supercritical carbon dioxide, allowing the *C. maxima* (pumpkin) seed kernels to be mixed with water at a 1:30 (w/v) ratio before protein extraction in an alkaline medium at pH 11.

During protein extraction, co‐extracted phenolic compounds could independently exert bioactive effects in vivo and in vitro, making it difficult to attribute observed activity to the peptide fraction alone. Therefore, downstream purification steps are incorporated into the pipeline to mitigate these issues. Initial purification is achieved by centrifugation and precipitation at the protein's isoelectric point. A study by Lin et al. (2024) precipitated *C. maxima* seed proteins at pH 5.3 using 1 N HCl after alkaline extraction. Similarly, Wani et al. (2011) used HCl at pH 4.5 to precipitate *Citrullus lanatus* seed proteins extracted with NaOH. However, further peptide enrichment is possible by fractionating the extract using chromatographic techniques, as discussed under the protein fragment purification and fractionation section.

### 2.2 Hydrolysis of the Extracted Proteins

BPs are generally encrypted within their parent proteins and become active only after proteolytic cleavage, either through in vivo enzymatic hydrolysis during food processing or during GI digestion (Rizzello et al. 2016). Conventionally, BP production relies on specific proteolytic enzymes or combinations of enzymes, often in conjunction with microbial fermentation.

#### 2.2.1 Enzymatic Hydrolysis

Enzymatic hydrolysis is influenced by critical parameters such as the type of enzyme, degree of hydrolysis (DH), enzyme–substrate ratio (E/S), and medium pH and temperature, where constant monitoring is essential (Silva et al. 2017). In most studies, enzymatic hydrolysis has been preferred over microbial fermentation due to its low reaction time, high predictability, and the possibility of applying more than one enzyme type simultaneously (Daliri et al. 2017). Enzymes used in these processes can originate from plants, fungi, microorganisms or animal digestive enzymes. Commonly applied proteases include bromelain, alcalase, papain, trypsin, chymotrypsin, pepsin, pancreatin, and thermolysin; however, their potency and efficacy vary with the protein source and the physicochemical properties of the substrate (Akbarian et al. 2022; Daliri et al. 2017). Lin et al. (2024) used the *C. maxima* seed extract, revealing that pepsin hydrolysate yielded the highest peptide content at 420.8 mg/g and significant in vitro ACE inhibition activity at 70.3%, surpassing papain and bromelain hydrolysates. Sequential or combined enzyme systems, rather than a single enzyme, often enhance hydrolysis efficiency by achieving a greater DH, which typically yields lower‐molecular‐weight (MW) peptides with elevated bioactivity. Arise (2016) used trypsin in a 1:100 E/S ratio for watermelon seed proteins, resulting in a DH of 26.3%, and the DH for alcalase in the same E/S ratio was 13.2%. However, Siddeeg et al. (2015) reported that the sequential use of trypsin and alcalase in a 1:100 E/S ratio for *C. moschata* seed proteins resulted in 28.2% of DH. Similarly, according to Vaštag et al. (2011), when alcalase and flavourzyme were individually used in 1:757 and 1:385 E/S ratios, respectively, for *Cucurbita pepo* (pumpkin) oil cake protein isolate, the DH were 53.2% and 37.2% for alcalase and flavourzyme fractions, respectively. When the two proteases were subsequently used in the E/S ratios of 1:250 for alcalase and 1:385 for flavourzyme, the oil cake protein isolate's DH was 69.3%. However, despite lower MW peptide fractions having a higher bioactive potential than higher MW fractions, the enzyme cocktail‐derived protein hydrolysate showed significantly lower in vitro antioxidant and ACE‐inhibitory activities than the alcalase hydrolysate. As stated by Ozuna and Leon‐Galvan (2017), peptide bioactivity tends to inversely correlate with molecular weight and chain length. Yet, during peptide synthesis or intracellular transport, proteases may cleave active sites of some peptides, reducing or eliminating their bioactive properties. As a result, some smaller peptides may degrade before exerting their actions, whereas larger MW peptides could resist degradation and retain their bioactive properties. Thus, the protease type (endopeptidase or exopeptidase), peptide AA composition, position of the active site, the biological mechanism by which the function is exerted, and the physicochemical parameters of the medium can affect peptide bioavailability and bioactivity.

#### 2.2.2 Microbial Fermentation

During fermentation, microorganisms produce proteolytic enzymes that metabolize protein substrates under optimal environmental conditions. *Bacillus* and *Lactobacillus* genera are recognized for their proteolytic capabilities (Aguilar‐Toala et al. 2017; Ahn et al. 2009; Rizzello et al. 2017; Gobbetti et al. 2000). For instance, Fideler et al. (2019) reported the use of lactic acid bacteria to ferment cucumber, which resulted in peptides with in vitro ACE‐inhibitory properties. Beyond bacterial fermentation, yeast and filamentous fungal enzymes from *Aspergillus* spp. also serve as valuable enzyme producers for BP generation (Ahn et al. 2009; Chaves‐Lopez et al. 2012; García‐Tejedor et al. 2015). Optimizing hydrolysis efficiency to produce smaller MW peptides often requires multiple fermentations, co‐culturing fungal and bacterial species, and/or supplementing with exogenous proteolytic enzymes (Akbarian et al. 2022).

Microbial fermentation is gaining interest over enzymatic hydrolysis to improve plant‐derived bioactive peptide production. Microbial strains can release a larger number of enzymes to generate short peptides from the plant matrices. Moreover, microbial fermentation lowers the downstream enzyme‐protein purification and separation costs, lowering the large‐scale peptide production costs (Nasri et al. 2022). Despite these advantages, microbial fermentation presents several limitations, such as the formation of undesirable compounds (formaldehyde, biogenic amines, and nitrites), temperature instability, and storage performance issues (Farid et al. 2024). To address these fermentation process‐related issues, incorporating innovative technologies such as ultrasonic, microwave, ohmic heating, and cold plasma can be used as a pretreatment step during solid‐state and submerged fermentation to enhance fermentation quality and yield while reducing reaction time (Valdés et al. 2022).

### 2.3 Protein Fragment Purification and Fractionation

Following hydrolysis, protein hydrolysates undergo fractionation to enrich biologically active peptides based on their molecular sizes. Membrane‐based techniques such as ultrafiltration are routinely used and act as molecular sieves but have limited selectivity. However, they are cost‐effective, highly productive and suitable for preliminary fractionation (Alavi and Ciftci 2023). The separated fractions are then tested in vitro and in vivo for their bioactivity (Vásquez‐Villanueva et al. 2018; Guo et al. 2015). Lin et al. (2024) used ultrafiltration with 10, 5, and 1 kDa MW cut‐off (MWCO) membranes for *C. maxima* seed protein hydrolysates prepared with papain, pepsin and bromelain enzymes. Peptide fractions below 1 kDa exhibited the highest ACE‐inhibitory and DPP‐IV potential, reinforcing that lower‐MW peptides generally exhibit greater bioactivity than parent hydrolysates (Lin et al. 2024).

Further fractionation is also possible with size‐exclusion chromatography (SEC) and reverse‐phase high‐performance liquid chromatography (RP‐HPLC). In SEC, molecules are separated by size; smaller molecules have higher retention times, whereas larger ones have lower retention times in the stationary phase. SEC has lower resolution than RP‐HPLC, making this approach ideal for the pre‐fractionation step of less complex samples. In contrast, RP‐HPLC separates molecules based on their hydrophobicity and can be used to analyze complex samples, such as peptides and organic compounds, for detailed characterization. RP‐HPLC‐derived fractions are commonly used in proteomic studies due to their compatibility with mass spectrometry (MS), whereas the phosphate buffer typically used in the SEC mobile phase renders the fractions incompatible with the MS system (Huang et al. 2018). However, SEC and RP‐HPLC fractions can be combined to increase the peptide detection and characterization efficiency. As an example, Sharma et al. (2014) reported using a Sephadex G‐75 column and HPLC to identify and characterize a novel antimicrobial peptide, hispidalin, from *Benincasa hispida* (wax gourd) seeds. Likewise, Wen et al. (2020) used Sephadex G‐15 followed by RP‐HPLC to isolate antioxidant peptides from watermelon seed protein < 1 kDa MW hydrolysate.

Overall, production of *Cucurbitaceae* peptides remains at an exploratory stage, where most studies rely on conventional enzymatic hydrolysis with in vitro assays, with little integration of fermentation, advanced extraction methods (such as ultrasound or microwave), or computational in silico prediction. Compared with legumes and cereals, methodological diversity is narrow and validation beyond the laboratory bench is virtually absent. This imbalance underscores the need for broader methodological application and cross‐species comparison across *Cucurbitaceae*.

## 3 Reported BPs From Family *Cucurbitaceae* and Their Functional Properties

The *Cucurbitaceae* family is one of the most extensively studied plant families for their nutritional properties and composition. Cucurbits are rich in compounds like alkaloids, terpenoids, tannins, and steroids. The presence of these molecules makes these crops enriched with pharmacological properties such as hypoglycemic, antihypertensive, antimicrobial, and anticarcinogenic activities. However, information on bioactive peptides from the family is limited to a few cucurbits, with tissue‐specific variation, except for the seeds, which are currently unknown. Table 2 summarizes the identified BPs from the family *Cucurbitaceae* and their functional roles based on currently reported studies. These functions will be discussed in detail in the following sections.

**TABLE 2:** Reported bioactivities of BPs sourced from the family *Cucurbitaceae* crops.

Protein/peptide source	Sequence	Molecular weight	Bioactivity	IC_50_/ED_50_/activity%	Study nature	References
*Cucurbita moschata* seed meal	SNHANQLDFHP PVQVLASAYR	< 3 kDa	Antihypertensive function by ACE‐inhibitory and ACE‐2 upregulating activities	172.07 90.69 μM	In vitro	Li et al. (2024)
*Cucurbita maxima* seed protein hydrolysates produced by papain, pepsin, and bromelain	Identification has not been completed	< 1, 1–5, and < 10 kDa	ACE‐inhibitory and DPP‐IV (dipeptidyl peptidase‐4) inhibitory action	Not available	In vitro	Lin et al. (2024)
*C. moschata* seed proteins (11S globulin and 2S albumin)	IAF		ACE‐inhibitory function	19.87 μM	In silico	Liang et al. (2021)
*Cucumis maderaspatensis* seed protein extract	Serine protease (CmSP)	~32 kDa	Anticoagulant and antiplatelet activities	Not available	In vitro	Sachin et al. (2021)
*Citrullus colocynthis* aerial parts of the plant	citcol‐1 to citcol‐8 (8 novel peptides)	3.5–4.5 kDa	Trypsin inhibitory function and antimicrobial function	Not available	In silico and in vitro	Shahin‐Kaleybar et al. (2020)
*Citrullus lanatus* seed protein hydrolysate using alcalase	RDPEER KELEEK DAAGRLQE LDDDGRL and GFAGDDAPRA	< 1 kDa	Antioxidant activity by protecting HepG2 cells from H_2_O_2_‐induced oxidative stress by inhibiting the ROS	0.216–0.435 (DPPH), 0.54–1.23 (ABTS) μM	In vitro	Wen et al. (2020)
*Momordica dioica* seed proteins	Identification has not been completed	< 30 kDa	Antiproliferative activity by inhibiting the growth of MCF‐7 cells	Not available	In vitro	Chandra et al. (2019)
*Cucumis sativus* fermented cucumber by lactic acid bacteria	IPP LPP VPP KP	0.42–0.49 mg/kg 0.30–0.33 mg/kg 0.32–0.35 mg/kg 0.93–1.5 mg/kg	Antihypertensive activity by Inhibition of ACE	Not available	In vitro	Fideler et al. (2019)
*C. lanatus* alcalase seed protein hydrolysate (WSPHS)	Identification has not been completed	< 1 kDa	Antioxidant activity by protecting RAW 264.7 cells from H_2_O_2_‐induced oxidative stress damage via activating the Nrf2/HO‐1 pathway	Not available	In vitro	Wen et al. (2019b)
*C. sativus* L. seed proteins	Identification has not been completed	—	Antibacterial effects against *E. coli*	Not available	In vitro	Al Akeel et al. (2018)
*Momordica charantia* seed proteins	R·DSDCLAQCICVDGHCG (BG‐4)	4 kDa	Anti‐inflammatory activity by reducing the production of pro‐inflammatory cytokines IL‐6 (interleukin‐6) and TNF‐α (tumor necrosis factor‐α)	Not available	In vitro	Jones et al. (2018)
*C. pepo* L. alcalase hydrolyzed pumpkin oil cake protein isolate (POCPH_1_)	Identification has not been completed	< 6.5 kDa	Antioxidant activity	Not available	In vitro	Nourmohammadi et al. (2017)
*C. lanatus* alcalase and tryptic hydrolysates of watermelon seed proteins	Identification has not been completed	—	Hypoglycemic effect by inhibiting the α‐amylase activity	Not available	In vitro	Arise (2016)
*M. charantia* seed proteins	R·DSDCLAQCICVDGHCG (BG‐4)	4 kDa	Anticancer activity by trypsin inhibitory function in HCT‐116 and HT‐29 colon cancer cells	134.4 (HCT), 217.0 (HT) μM	In vitro	Dia and Krishnan (2016)
*Momordica cymbalaria* fruit extract	Mcy Protein (MCP)	—	Hypoglycemic function by inhibiting the activity of α‐glucosidase and aldose reductase enzymes	Not available	In vivo and in vitro	Marella et al. (2016)
*M. charantia* thermolysin hydrolysate of seed proteins	VSGAGRY (VY‐7) VDSDVVKG (VG‐8)	< 3 kDa	Antihypertensive function through ACE inhibition	8.64 and 13.30 μM	In silico, in vitro and in vivo	Priyanto et al. (2015)
*Cucumis melo* (Seinat) trypsin + pepsin seed protein hydrolysate	Identification has not been completed	3–5 kDa	Antioxidant activity	Not available	In vitro	Siddeeg et al. (2015)
*C. moschata* seed meal hydrolysate using alcalase, neutrase, flavourzyme, and protamex	Sequencing has not been done. AA composition of the hydrolysates has been determined	0.18–1 kDa^a^ 1–5 kDa^a^ > 10 kDa^a^ 0.18–1 kDa^a^	Antioxidant activity	Not available	In vitro	Venuste et al. (2013)
*Benincasa hispida* seed proteins	SDYLNNNPLFPRYDIGNVELSTAYRSFANQKAPGRLNQNWALTADYTYR (Hispidalin)	~6 kDa	Antimicrobial activity by broad and potent inhibitory effects against various human bacterial and fungal pathogens and antioxidant activity	70.8% (DPPH), 69.5% (lipid peroxide inhibition)	In vitro	Sharma et al. (2014)
*C. pepo* L. alcalase and flavourzyme hydrolysates of pumpkin oil cake protein isolate (PuOC PI)	Identification has not been completed	< 15 kDa	Antioxidant activity and ACE‐inhibitory function	Not available	In vitro	Vaštag et al. (2011)
*Momordica cochinchinensis* seed proteins	MCoCC‐1 (33 AA) MCoCC‐2 (32 AA)	3.287 kDa 3.169 kDa	Anticancer activity against the human melanoma cell line (MM96L)	Not available	In vitro	Chan et al. (2009)
*M. charantia* fruit flesh proteins	MC2‐1‐5 (GHPYYSIKKS)	3.4 kDa	Hypoglycemic effect by lowering the blood glucose level (BGL) through enhancing glucose utilization	Not available	In vivo	Yuan et al. (2008)
*C. pepo* seed proteins	Identification has not been completed	—	Antiperoxidative activity by alleviating CCl4 intoxication in acute liver injury	Not available	In vivo	Nkosi et al. (2006b)
*M. charantia* fruit pulp	Identification has not been completed	—	Hypoglycemic effect as a slow‐acting insulin secretagogue and insulinomimetic agent upon subcutaneous administration into streptozotocin‐induced diabetic rats	Not available	In vivo	Yibchok‐anun et al. (2006)
*C. moschata* cv. seed proteins	PQRGEGGRAGNLLREEQEI (cucurmoschin)	9 kDa	Antifungal activity against *Fusarium oxysporum*, *Botrytis cinerea* and *Mycosphaerella arachidicola*	1.2 μM	In vitro	Wang and Ng (2003)
*C. maxima* seed proteins	P2249 P4650 Napin‐like complex P11696 (large and small subunits)	2.2 kDa 4.6 kDa 11.7 kDa	Antifungal effect on different phytopathogenic fungi	Not available	In vitro	Vassiliou et al. (1998)
*M. charantia* seed proteins	Polypeptide‐p (166 AA residues)	~11 kDa	Hypoglycemic activity upon subcutaneous administration	Not available	In vivo	Khanna et al. (1981)

*Note:* DPPH—DPPH radical scavenging activity; ABTS—ABTS radical scavenging activity.

^a^ Predominant MW peptide fractions of different hydrolysates of *Cucurbita moschata* seed meal.

### 3.1 Antihypertensive Activity

Hypertension affects over 1 billion people worldwide and contributes to more than 8 million deaths annually (Du and Li 2022). The cornerstone mechanism for hypertension is the ACE‐angiotensin conversion, where ACE converts angiotensin I into angiotensin II, which positively correlates with elevated blood pressure (hypertension) (Hartmann and Meisel 2007). Suppressing ACE activity is a key therapeutic strategy for hypertension, and several cucurbits have been reported to have ACE‐inhibitory peptides.

Four short peptides, 2–3 residues in length (IPP, LPP, VPP, and KP), have been identified by Fideler et al. (2019) from fermented cucumber with ACE‐inhibitory properties. The fourth peptide was found to have a 3–5‐fold greater abundance in fermented cucumber than in acidified cucumber, highlighting the role of microbial enzymes in generating low‐MW peptides with functional importance. However, the previously reported IPP and VPP antihypertensive peptide levels were not increased to clinically relevant concentrations in fermented cucumber, and the cumulative antihypertensive effect of the detected peptides was not assessed in that study.

Li et al. (2024) also reported *C. moschata* seed meal as a promising source of antihypertensive peptides. This study isolated SNHANQLDFHP and PVQVLASAYR peptides from pumpkin seed meal using neutrase, with dual functions: ACE inhibition and ACE2 upregulation. These peptides were predicted to interact with ACE through hydrogen bonds and hydrophobic interactions, thereby exerting ACE‐inhibitory activity. The two peptides were also tested for stability during GI digestion, in which SNHANQLDFHP showed a 14.03% increase in functionality compared to pre‐digestion levels. Since the simulated conditions for GI digestion may differ from the actual physiological conditions, and the structural changes of the peptides after digestion were not analyzed, further in vivo studies are required to confirm the functionality and stability of these peptides during GI digestion. As highlighted by Lin et al. (2024), *C. maxima* seed protein hydrolysates by papain, bromelain, and pepsin all exhibited ACE‐inhibitory activity. Functionality was assessed at the fraction level, with the highest ACE‐inhibitory rate (83.27%) recorded for the pepsin hydrolysate fraction with an < 1 kDa MW. Although a simulated GI stability test was also conducted to confirm the enhanced functionality of the pepsin hydrolysate, the absence of in vivo evaluations limits the strength of this validation.

### 3.2 Antioxidant Activity

Oxidative stress arises from imbalances in the production and neutralization of reactive oxygen and nitrogen species, leading to lipid, protein, and DNA damage and contributing to chronic diseases (Forman and Zhang 2021; Lopez‐Garcia et al. 2022). Cucurbits have been identified as rich sources of antioxidative compounds, including peptides capable of radical scavenging, metal chelation, and lipid peroxidation inhibition (Xiong 2010). A study by Wen et al. (2020) identified five peptides with potential antioxidative properties from watermelon seed proteins. The peptide sequence RDPEER showed the highest antioxidative potential among the peptides KELEEK, DAAGRLQE, LDDDGRL, and GFAGDDAPRA, which is the smallest‐MW peptide. The antioxidant properties of the identified peptides were evaluated using 2,2‐diphenyl‐1‐picrylhydrazyl (DPPH) radical scavenging capacity, 2,2′‐azino‐bis(3‐ethylbenzothiazoline‐6‐sulfonic acid) (ABTS) radical scavenging capacity, and oxygen radical absorbance capacity assays, with IC_50_ values of 0.216–0.435, 0.54–1.23, and 82.36–130.67 μM, respectively. The proportion of antioxidant AA residues in these peptides ranged from 16.7 to 70, contributing positively to their in vitro antioxidant potential. Moreover, Wen et al. (2019) reported that watermelon seed protein hydrolysate with < 1 kDa MW exhibits strong antioxidant potential, protecting H_2_O_2_‐induced oxidative‐stressed macrophage cell lines by activating the Nrf2/HO‐1 pathway and preventing cell damage, supporting the notion that the watermelon seed proteins possess significant antioxidant properties; however, further in vivo validation is required.

Similarly, Sharma et al. (2014) analyzed *Benincasa hispida* seeds and isolated a novel antioxidant peptide, hispidalin, which is also an antimicrobial peptide, with 49 AA residues and a MW of 5.7 kDa. This peptide has been identified as an excellent source of antioxidative potential with 70.8% DPPH free radical‐scavenging capacity and 69.5% lipid peroxide inhibition. The composition analysis of hispidalin revealed the presence of three serine, five tyrosine, and three threonine residues with hydroxyl groups, contributing to its antioxidant properties by free radical‐scavenging activity. Nourmohammadi et al. (2017) examined *C. pepo* oil cake protein isolate (POCPI) hydrolyzed by alcalase and trypsin, showing enhanced antioxidative potential compared to the POCPI. However, among the two POCPI hydrolysates, the alcalase fraction has been identified with higher DPPH free radical scavenging capacity and ferrous ion chelating capacity. Additionally, this alcalase hydrolysate of POCPI has been reported with broader thermal stability, even at 100°C and pH stability at pH ranging from 3 to 7, with no significant change in its antioxidative potential.

### 3.3 Antimicrobial Activity

**FIGURE 2:**
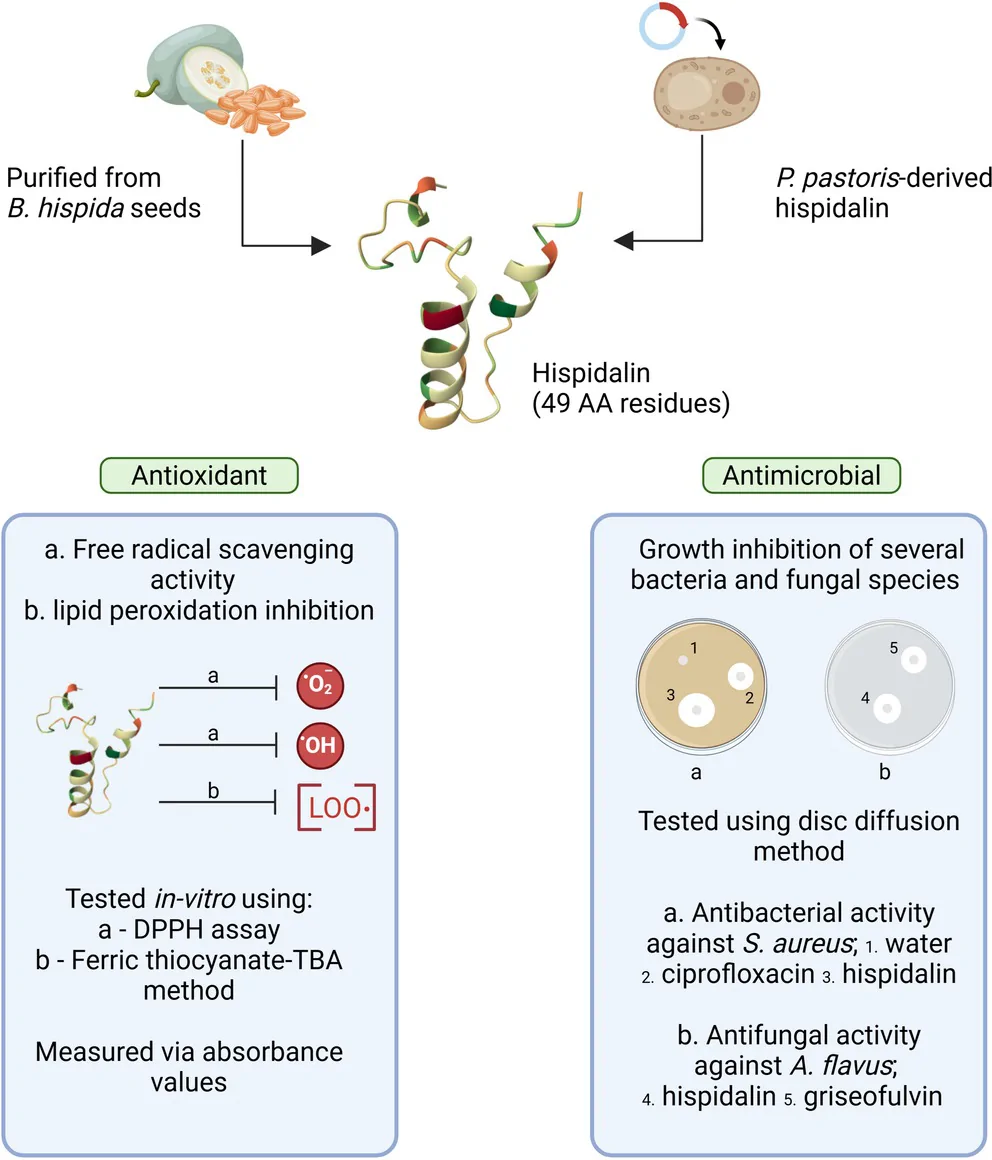
Antioxidant and antimicrobial functions of hispidalin. Hispidalin (49 AA residues, 5.7 kDa) is derived from *Benincasa hispida* (wax gourd) seed protein hydrolysate and recombinantly produced via *Pichia pastoris*. This peptide exhibits in vitro antioxidant and antimicrobial effects. Hispidalin's 3D structure was predicted from PEP‐FOLD3; colors in the peptide model represent different levels of hydrophobicity of the structural residues: In red, the highest hydrophobicity and in green the lowest. The inhibition zone sizes indicate relative antimicrobial activity and are not to scale. This image was created using BioRender.

Antimicrobial peptides (AMPs) are essential components in plant defense mechanisms and provide a wide array of protection against fungi, bacteria, and virus infections. Antibacterial activity of seed proteins of *Cucumis sativus* (cucumber) against *Escherichia coli* , *Staphylococcus aureus* , *Proteus vulgaris* , and *Pseudomonas aeruginosa* has been reported by Al Akeel et al. (2018). It had the highest antibacterial potential against *E. coli* , which makes *C. sativus* seeds a possible source for new AMP discoveries. Sharma et al. (2014) tested hispidalin against a panel of microorganisms, including bacterial strains ( *E. coli* , *P. aeruginosa* , *S. enterica* , *S. aureus* , *Bacillus cereus* ) and fungal species (*Aspergillus flavus*, *Penicillium chrysogenum*, *Fusarium solani*, *Curvularia geniculata*, *Colletotrichum gloeosporioides*). Hispidalin displayed the highest inhibition against *S. enterica* and the weakest against *P. aeruginosa* among the bacterial species. Additionally, the findings indicate the highest inhibition level against *A. flavus* and the lowest against *C. geniculata* among the fungal species. Hispidalin from *Benincasa hispida* was identified as a potent AMP, and its inhibitory activities were significantly comparable to those of commercially available antimicrobial agents. Figure 2 summarizes the functional mechanism of hispidalin as a peptide with potent bioactive properties. Moreover, Wang and Ng (2003) reported a novel antifungal peptide, cucurmoschin, from *C. maxima* (black pumpkin) seeds with a molecular weight of 8 kDa and an IC_50_ of 1.2 μM of translation inhibitory activity. Cucurmoschin exhibited antifungal activity against *Botrytis cinerea*, *Fusarium oxysporum*, and *Mycosphaerella oxysporum* by inhibiting mycelial growth. The authors suggested that the antifungal potency of cucurmoschin may stem from the abundance of arginine, glutamate, and glycine at the N‐terminus of the peptide. However, this functional validation remains limited to in vitro conditions.

### 3.4 Anticancer Activity

Plant‐based therapeutics in chemoprevention and therapy are an emerging field of science. The *Cucurbitaceae* family contains bioactive compounds with higher anticancer potential. Genus *Momordica* has been identified as a potential source of anticancer peptides. For example, Chandra et al. (2019) reported that the *Momordica dioica* (spiny gourd) seed proteins possess anticancer properties, demonstrated by their ability to inhibit the growth of MCF‐7 cells by 93.2%. However, peptide characterization was not performed in that study, and the in vitro analysis was conducted using the < 30 kDa MW peptide fraction. Furthermore, Chan et al. (2009) revealed the anticancer potential of *Momordica cochinchinensis* (gac) by isolating two novel peptides, MCoCC‐1 and MCoCC‐2. These peptides were identified as highly homologous to each other and consist of 33 and 32 AAs, respectively. Of the two peptides, MCoCC‐1 was identified with the highest toxicity against a human melanoma cell line (MM96L) and no hemolytic effect against human erythrocytes. Another study by Dia (2021) reported a novel anticancer peptide named BG‐4 from *Momordica charantia* (bitter gourd). The peptide BG‐4 has been reported to have potent trypsin‐inhibitory potential, which is 8.6 times higher than that of soybeans. This higher trypsin inhibitory activity in BG‐4 could be the reason for its characteristic toxicity against human colon cancer cells, promoting their apoptosis. According to the study results, the apoptosis rate of colon cancer cells went from 8.5% to 31.9% after 16 h of treatment with BG‐4.

### 3.5 Antidiabetic Activity

Peptides from *Momordica* sp., from the *Cucurbitaceae* family, have been documented for their antidiabetic and hypoglycemic properties. For instance, Yuan et al. (2008) reported the hypoglycemic activity of the *M. charantia* L. MC1‐2‐5 (GHPYYSIKKS) peptide with 3.4 kDa MW. Orally administered MC1‐2‐5 reduced blood glucose levels by 61.7% and 69.2% in 2 and 4 h, respectively, in alloxan‐induced diabetic mice. Likewise, Khanna et al. (1981) identified a hypoglycemic polypeptide from *M. charantia* L., named polypeptide‐p (~11 kDa), wherein subcutaneous administration demonstrated potent in vivo hypoglycemic effects in humans, langurs, and gerbils. Similarly, Marella et al. (2016) reported that the Mcy protein (MCP) isolated from *Momordica cymbalaria* treatment of 2.5 mg/kg exhibited antihyperglycemic and antioxidant activities in streptozotocin‐induced diabetic rats. MCP normalized key carbohydrate metabolizing enzymes (hexokinase, glucose‐6‐phosphatase, and glucose‐6‐phosphate dehydrogenase). Compared to the other biological functionalities, the antidiabetic properties of *Cucurbitaceae*, most notably in bitter gourd, have been validated in vivo and used in traditional medicine in several Asian and African countries (Selvakumar et al. 2017). Furthermore, MCP has exhibited its antidiabetic function in vitro by inhibiting the activity of α‐glucosidase and aldose reductase enzymes. MCP reduced oxidative stress by suppressing ROS and lipid peroxidation. Arise (2016) reported the hypoglycemic effect of alcalase and tryptic hydrolysates of watermelon seed proteins. These hydrolysates exhibited in vitro hypoglycemic effects by inhibiting the α‐amylase activity, and the values for α‐amylase inhibition by the alcalase and tryptic hydrolysates were recorded as 86% ± 4% and 83% ± 4%, respectively.

## 4 Industrial Applications of Plant BPs From *Cucurbitaceae* and Possible Challenges

**FIGURE 3:**
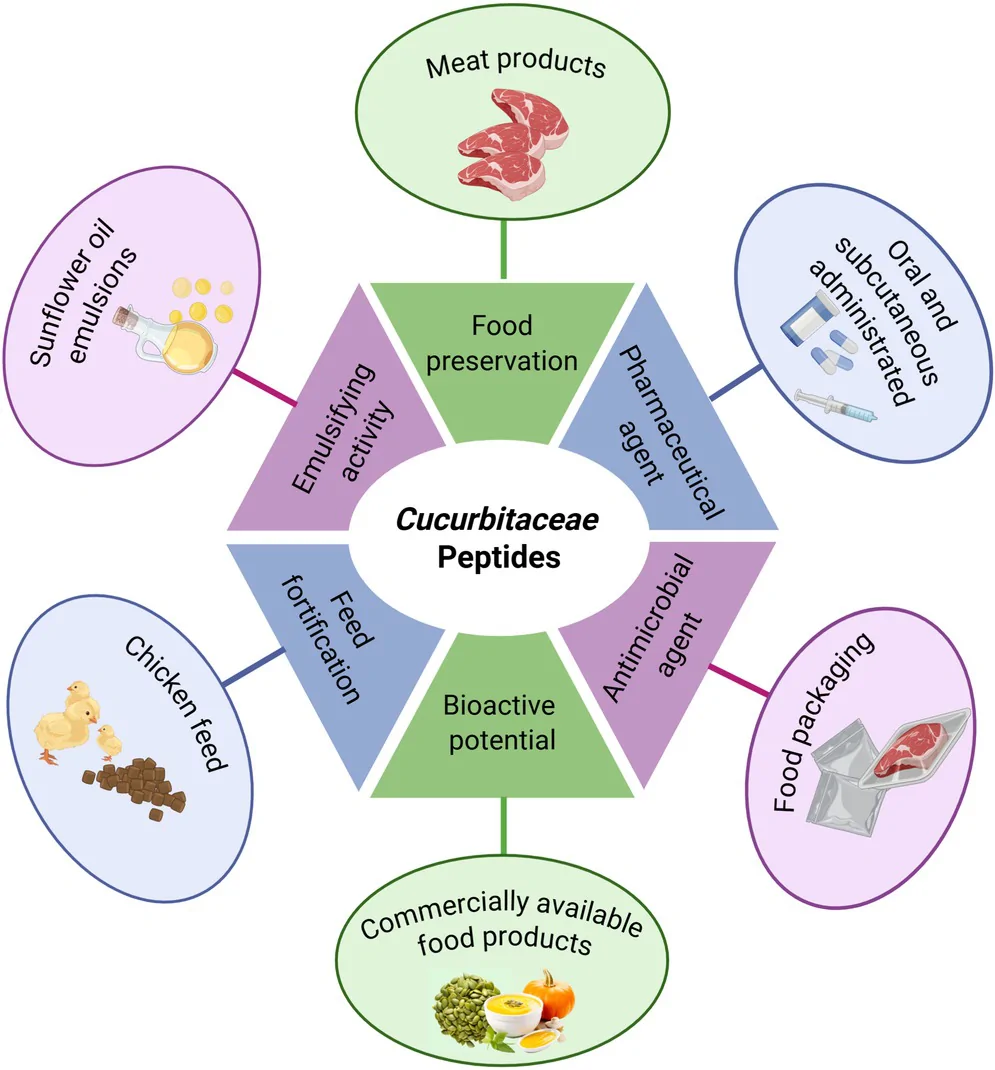
Industrial usage of *Cucurbitaceae* peptides. Cucurbit peptides are utilized in the food, feed, and pharmaceutical industries due to their potential bioactive properties, including antimicrobial, antioxidant, and antidiabetic effects. The image was created using BioRender.

Among the cucurbit peptides with proven bioactive properties, as discussed in the previous section, several have been utilized primarily in food, feed, and pharmaceutical industries. Figure 3 highlights the industrial utilization of *Cucurbitaceae* peptides according to the current literature.

### 4.1 Food and Feed Industry

Plant‐derived peptides have attracted growing interest in the food industry due to their bioactive properties to enhance the nutritional benefits of food (Fan et al. 2022). The techno‐functional properties of plant‐derived BPs, like emulsifying ability, water–oil retention capacity, flavor‐enhancing quality, and acidity‐balancing properties, underscore their importance in achieving desired food quality (Fan et al. 2022). *Cucurbitaceae* fruits are popular food sources used in salads and curries and are occasionally mixed with dairy to prepare yoghurt, soups, etc. Additionally, seeds from a few different cucurbits, such as watermelon, pumpkin, and melon, are available as a popular snack. However, limited information is available on the bioactive peptide composition and functional properties of these available products compared to other plant‐based products available in the commercial market. Bucko et al. (2015) identified 12S globulins (cucurbitins, ~25 kDa) as the predominant fraction in *C. pepo* seed protein isolate (PSPI), noting their natural emulsifying properties in sunflower oil emulsions. The protein and peptides' emulsifying activity is governed by the amphiphilicity, length, and interfacial conformation of the AA chains (García‐Moreno et al. 2023). Subsequently, Bucko et al. (2016) revealed that the pepsin hydrolysate of the pumpkin seeds (PSPH) possesses relatively higher emulsifying properties than PSPI, regardless of the ionic strength at all the tested pH points in 20% sunflower oil emulsions. Separately, hispidalin from *Benincasa hispida* has been used as a food preservative agent in a pork‐meat model, which has contributed to maintaining pork quality by enhancing free‐radical scavenging activity and preventing bacterial growth when stored at 4°C (Meng et al. 2020).

The use of plant‐derived peptides from *Cucurbitaceae* in the feed industry was investigated by Nguyen et al. (2019). The authors used watermelon rind powder (WRP) at a concentration of 9% to fortify a commercially available starter diet for 3‐ to 15‐day‐old chickens, resulting in an elevation of plasma L‐citrulline levels. This study demonstrated that watermelon rind is a natural source of L‐citrulline for animal feed, offering natural hypothermic benefits against heat stress, a growing concern within the poultry industry. However, this level of fortification with WRP was insufficient to induce a hypothermic effect in chicks, despite elevating plasma L‐citrulline, leaving the question of how much WRP is required in feed fortification to induce a hypothermic effect. In addition, the use of plant‐derived AMPs in animal feed is also a popular approach in the animal feed industry (Silveira et al. 2021).

### 4.2 Pharmaceutical Industry

The reported bioactivities of plant‐derived peptides, including antioxidant, antidiabetic, and antihypertensive properties, have made these BPs a potential source for pharmaceutical production, especially AMPs, as these molecules demonstrate a broad range of effects against fungi, viruses, bacteria, and even parasites (de Souza Candido et al. 2014). Moreover, hispidalin, the AMP isolated from wax gourd, has been reported to possess antifungal, antibacterial, and antioxidant properties, with significant DPPH free‐radical scavenging activity (Sharma et al. 2014). Hispidalin exemplifies dual functionality, showing antimicrobial and antioxidant activity. Consequently, hispidalin can be used as a preservative in pharmaceutical products.

Peptides from *M. charantia* have also gained pharmaceutical relevance. Small peptides (< 10 kDa), MC6.1, MC6.2, and MC6.3, have been recognized for their hypoglycemic effects upon oral administration and were granted a European patent in 2008 (EP 1005274 B1) (Nag et al. 2008). Continuing the trend of exploring the pharmaceutical applications of BPs, Yibchok‐anun et al. (2006) reported that the subcutaneous administration of *M. charantia* protein extract from fruit pulp to streptozotocin‐induced diabetic rats had a significant hypoglycemic effect as a slow‐acting insulin secretagogue and insulinomimetic agent. As mentioned by Sosalagere et al. (2022), bitter gourd is called the ‘plant insulin’ due to its anti‐diabetic effects, which makes bitter gourd a promising source of BPs in drug development.

### 4.3 Challenges Associated With Plant‐Based Peptides

Despite their broad potential in food, feed, and pharmaceutical applications, the industrial utilization of plant‐derived peptides faces several major challenges, primarily related to taste, stability, and scalability. The bitter taste of many plant‐derived BPs limits consumer acceptance, particularly in food and oral supplement applications. Peptides' bitterness is influenced by composition, hydrophobicity, and structural conformation. Hydrophobic amino acids such as phenylalanine, arginine, isoleucine, leucine, tryptophan, and tyrosine are often responsible. The food industry uses debittering processes—such as solvent extraction, ultrafiltration, chromatographic separation, and isoelectric precipitation—to eliminate, mask, or reduce the bitter‐tasting AAs from the BPs (Liu et al. 2022). These separation methods are easy to perform, can effectively remove the bitterness from the peptides, but increase processing costs and can lead to degradation of some of the BPs with higher bioactive potential (Chakrabarti et al. 2018). According to Chakrabarti et al. (2018), bitterness positively correlates with the MW of the peptides; thus, additional enzyme treatments would be beneficial for hydrolyzing the BPs. Lei et al. (2021) reported using an arginine aminopeptidase from *Bacillus axarquiensis* on the pumpkin seed protein isolates (PSPI) hydrolyzed by alcalase, papain, and flavourzyme to reduce bitterness. The bitter intensity score of all three hydrolysates with the bacterial peptidase was reduced compared to that of hydrolysates without the bacterial peptidase.

The other major challenge associated with the industrial utilization of BPs is the lack of stability. As mentioned by Fan et al. (2022), BPs can lose their functional properties due to excess heating, which is unavoidable during large‐scale production. Furthermore, due to their fragile nature and low stability, which are characteristic of most plant‐derived BPs, they often break down in the digestive system before being absorbed systemically into the body. This can be minimized by encapsulating BPs using nanomaterials, polysaccharides, or liposomes. Like the food and feed industries, the pharmaceutical industry faces similar challenges, such as the bitter taste when administered orally and the low stability of plant‐derived BPs.

As highlighted by de Souza Candido et al. (2014), natural peptide yields are often low. Scaling up production requires substantial starting material, purification steps, extraction solvents, and other resources, making the process expensive and environmentally unsustainable. Chemical synthesis of BPs has been implemented as an alternative, as this approach allows the production of pure, well‐defined peptide forms. Solid‐phase peptide synthesis remains the most widely applied method in many BP discovery studies. However, it has been identified as ineffective for large‐scale production due to the costs of peptide synthesis, especially when peptides are cyclized with disulfide bonds and PTMs (de Souza Candido et al. 2014). Furthermore, no studies have reported the chemical synthesis of *Cucurbitaceae*‐derived peptides. Recombinant DNA technology has been identified as an efficient alternative to produce large amounts of BPs in a shorter time at a lower cost via cloning the exogenous peptide‐encoding gene in a selected vector. As reported by Tavares et al. (2012) *E. coli* has been used as the vector in producing Pg‐AMP1, a powerful antibacterial peptide against multiple gram‐positive and gram‐negative bacteria discovered in guava seeds. However, host selection critically affects peptide yield and activity; for instance, recombinant hispidalin (rHispidalin) produced using *E. coli* lacked the expected antibacterial efficacy against both Gram‐positive and Gram‐negative bacteria, whereas *Pichia pastoris*‐derived hispidalin retained strong bactericidal activity, affirming its suitability as a potent vector in large‐scale hispidalin production (Meng et al. 2019).

## 5 Future Perspectives

Plants continue to represent a sustainable and abundant source of exogenous bioactive peptides (BPs) with promising nutraceutical and therapeutic applications. Within this context, *Cucurbitaceae* crops have been consumed since their domestication yet hold underexplored potential. Over the past decades, plant‐derived peptide production has expanded at both laboratory and industrial scales, with many peptides showing promising bioactivities. However, current evidence remains limited: most findings are derived from in vitro assays, whereas in vivo animal studies and clinical trials are limited. Establishing the bioavailability, pharmacokinetics, and safety of these peptides through human trials will be crucial for translational progress. As *Cucurbitaceae‐*derived peptides currently lack any regulatory precedent, they would likely require classification as novel food ingredients—a pathway demanding extensive toxicological, purity, stability, and bioactivity‐consistent data prior to market approval. Emerging computational tools such as machine learning (ML) and artificial intelligence (AI) can accelerate peptide discovery, overcoming the labor‐intensive, costly, and time‐consuming limitations of conventional approaches. Integrating these computational pipelines with proteogenomics—combining transcriptomic and genomic mining with mass spectrometry‐based proteomics—can generate peptide sequence databases that expand discovery into understudied cucurbit species, beyond the few economically important crops that dominate current research. This approach may reveal structurally unique peptides with novel bioactivities.

Future research should also address tissue specificity and processing effects, since most studies remain seed‐centric. Exploring underutilized tissues such as leaves, rinds, and peels could not only diversify bioactive profiles but also contribute to circular economy approaches, turning agricultural by‐products into high‐value peptide sources. This valorization has both economic and sustainability advantages, reducing waste streams. Another major gap lies in the stability, safety, and delivery systems for *Cucurbitaceae*‐derived peptides. Studies should prioritize bioavailability, gastrointestinal stability, and formulation strategies (e.g., encapsulation, nano‐delivery systems) to bridge the laboratory‐to‐market gap.

Finally, *Cucurbitaceae* peptides should be evaluated comparatively with other major plant sources (legumes, cereals). Highlighting where cucurbits excel—such as structural novelty, diversity of peptide motifs, or bioactivities less common in other crops—will help justify their inclusion in the future functional food and nutraceutical markets. By critically evaluating current findings and highlighting translational gaps, this review positions *Cucurbitaceae* as an emerging yet underutilized source of bioactive peptides, warranting deeper investigation for nutraceutical and pharmaceutical development.

## Author Contributions

**Himani R. Kekiri Obada Gamage:** conceptualization, visualization, writing – review and editing, writing – original draft. **Mahya Bahmani:** writing – review and editing, supervision. **Utpal Bose:** writing – review and editing, supervision. **Mekhala Vithana:** writing – review and editing, supervision. **Michelle L. Colgrave:** writing – review and editing, supervision, funding acquisition. **Angéla Juhász:** conceptualization, writing – review and editing, project administration.

## Funding

This project was supported by the Australian Research Council (ARC) Centre of Excellence for Innovations in Peptide and Protein Science (grant number CE200100012) and Edith Cowan University, Western Australia.

## Ethics Statement

This manuscript is a narrative review that synthesizes and discusses information from previously published studies, which are appropriately cited. As it is not original research with human participants, animals or personal data, it has not been submitted for ethics committee approval. All the contributors to the review confirm adherence to academic integrity standards, including proper attribution of sources and absence of plagiarism.

## Conflicts of Interest

The authors declare no conflicts of interest.

## Data Availability

Data sharing is not applicable to this article as no datasets were generated or analyzed during the current study.
